# The effects of enhanced methionine synthesis on amino acid and anthocyanin content of potato tubers

**DOI:** 10.1186/1471-2229-8-65

**Published:** 2008-06-12

**Authors:** Gábor Dancs, Mihály Kondrák, Zsófia Bánfalvi

**Affiliations:** 1 Agricultural Biotechnology Center, P.O. Box 411, H-2101 Gödöllõ, Hungary

## Abstract

**Background:**

Potato is a staple food in the diet of the world's population and also being used as animal feed. Compared to other crops, however, potato tubers are relatively poor in the essential amino acid, methionine. Our aim was to increase the methionine content of tubers by co-expressing a gene involved in methionine synthesis with a gene encoding a methionine-rich storage protein in potato plants.

**Results:**

In higher plants, cystathionine γ-synthase (CgS) is the first enzyme specific to methionine biosynthesis. We attempted to increase the methionine content of tubers by expressing the deleted form of the *Arabidopsis CgS *(*CgS*_*Δ*90_), which is not regulated by methionine, in potato plants. To increase the incorporation of free methionine into a storage protein the *CgS*_*Δ*90 _was co-transformed with the methionine-rich *15-kD β-zein*. Results demonstrated a 2- to 6-fold increase in the free methionine content and in the methionine content of the zein-containing protein fraction of the transgenic tubers. In addition, in line with higher methionine content, the amounts of soluble isoleucine and serine were also increased. However, all of the lines with high level of CgS_Δ90 _expression were phenotypically abnormal showing severe growth retardation, changes in leaf architecture and 40- to 60% reduction in tuber yield. Furthermore, the colour of the transgenic tubers was altered due to the reduced amounts of anthocyanin pigments. The mRNA levels of phenylalanine ammonia-lyase (*PAL*), the enzyme catalysing the first step of anthocyanin synthesis, were decreased.

**Conclusion:**

Ectopic expression of CgS_Δ90 _increases the methionine content of tubers, however, results in phenotypic aberrations in potato. Co-expression of the 15-kD β-zein with CgS_Δ90 _results in elevation of protein-bound methionine content of tubers, but can not overcome the phenotypical changes caused by CgS_Δ90 _and can not significantly improve the nutritional value of tubers. The level of *PAL *mRNA and consequently the amount of anthocyanin pigments are reduced in the CgS_Δ90 _transgenic tubers suggesting that methionine synthesis and production of anthocyanins is linked.

## Background

**Figure 1:**
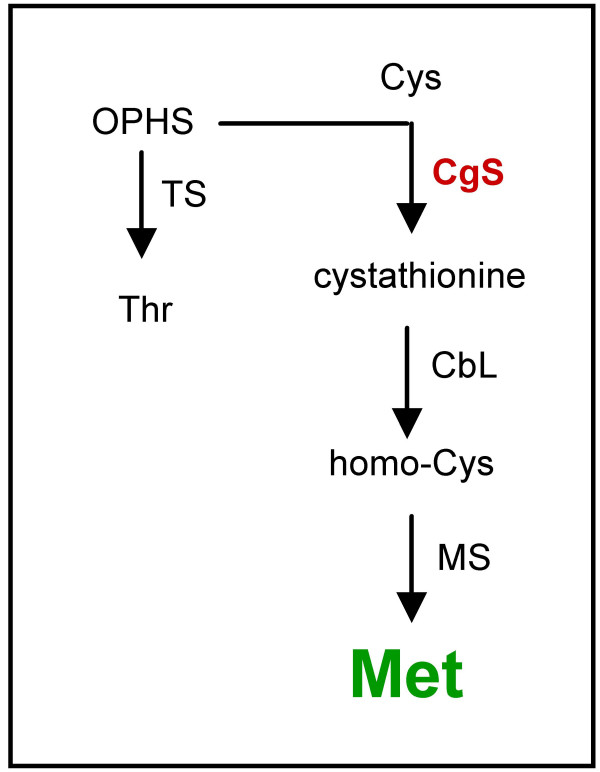
**Pathway of methionine synthesis**. OPHS, *O*-phospho-homoserine; TS threonine synthase; CgS, cystathionine γ-synthase; CbL, cystathione β-lyase; MS, methionine synthase.

Methionine is synthesised via a branched pathway (Figure 1) with complex regulatory circuit. In plants, the branch point intermediate of methionine synthesis is *O*-phospho-homoserine (OPHS), which is a common substrate for both threonine synthase (TS) and cystathionine γ-synthase (CgS). OPHS is directly converted to threonine by TS, while methionine is synthesised in three steps. Condensation of cysteine and OPHS is catalysed by CgS resulting in cystathionine, which is subsequently converted to homocysteine by cystathione β-lyase, and methionine by methionine synthase [1].

CgS and TS compete for the common substrate OPHS. It is an interesting feature of plants that TS is activated through *S*-adenosyl-L-methionine (SAM), a metabolite derived from methionine [2]. TS is the major control point for methionine biosynthesis [3]. Reduction of TS activity to 6% of the wild-type potato by antisense inhibition led to a 2- to 240-fold increase in methionine content in leaves and a 2- to 30-fold increase in methionine content in tubers. Strong reduction of TS activity and/or significant accumulation of methionine, however, were accompanied by severe phenotypic changes and acute reduction in tuber yield [4].

An alternative possibility to increase methionine content of plants is the over-expression of CgS. Constitutive over-expression of CgS in *Arabidopsis *caused an 8- to 20-fold elevation of the methionine content. However, the increased methionine level was observed only in seedling tissues and flowers, siliques, and roots of mature plants, but not in mature leaves [5]. Transgenic tobacco, potato, and alfalfa plants over-expressing the *Arabidopsis *CgS also had higher levels of soluble methionine [6-8]. Using this approach, the increase of methionine levels in tubers of transgenic potato lines was 6-fold compared to those in wild-type potato plants [7].

Studies in *Arabidopsis *revealed that the activity of CgS, which is located in the chloroplast, is regulated at both the transcript and protein levels indirectly by methionine via SAM. CgS contains approximately 100 amino acids in the N-terminal that controls CgS activity [9-11]. Recently, another *CgS *transcript was found in *Arabidopsis*, which contains an internal 90-nucleotide deletion at the N-terminal region (*CgS*_*Δ*90_). It was demonstrated that transgenic tobacco plants over-expressing CgS_Δ90 _display a significantly higher level of methionine than plants over-expressing the full-length CgS [12].

In potato, CgS activity is not modulated by methionine via SAM despite the fact that the N-terminal region of potato CgS is very similar to the regulatory region of *Arabidopsis *CgS [4,13]. Over-expression of the endogenous CgS in potato resulted in 2.7-fold elevated CgS activities but unchanged methionine levels [14].

Soluble amino acid levels of crops can be lost in food processing or cooking. Such losses can be minimised if the amino acids are incorporated in stable proteins. We attempted, therefore, to increase the protein-bound methionine content of potato by combined expression of CgS_Δ90 _and the methionine-rich protein, 15-kD β-zein. Effects of the transgenes on the phenotype and yield of the plants as well as on the amino acid composition and protein content of tubers are reported here.

## Results

### Isolation of potato plants expressing CgS_Δ90 _and both CgS_Δ90 _and the 15-kD β-zein genes

**Figure 2:**
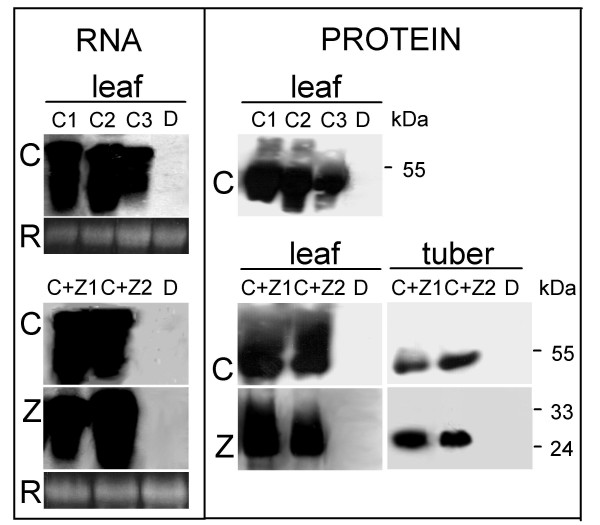
**Molecular characterisation of transgenic lines**. RNA- and protein-blot analysis of the *CgS*_*Δ*90 _transgenic lines C1, C2, C3, and *CgS*_*Δ*90_+*zein *double-transgenic lines C+Z1 and C+Z2. C, CgS_Δ90_; Z, zein; D, non-transformed control Désirée; R, ethidium-bromide-stained rRNA bands shown as loading controls. To detect CgS_Δ90 _and the 15-kD β-zein, proteins of the water-soluble and the ethanol-soluble fractions, respectively, were separated by SDS-PAGE and analysed using antibodies against the 3HA peptide. Molecular mass standards were included in the gels, and the sizes of the relevant markers are indicated in kDa.

To obtain potato plants expressing CgS_Δ90 _we transformed the cultivar Désirée with a construct containing the *CgS*_*Δ*90 _cDNA driven by the constitutive *CaMV 35S *promoter, and fused to a chloroplast transit peptide at the 5' end for targeting, and three copies of heamagglutinin (*3HA*) at the 3' end for immunological detection [12]. Thirty kanamycin-resistant lines were obtained. PCR analysis using *CgS*_*Δ*90_-specific primers detected the presence of the transgene in 27 lines. The expression levels of *CgS*_*Δ*90 _were determined at both transcript and protein levels by RNA- and protein-blot analysis using anti-HA monoclonal antibody for detection of the CgS_Δ90 _fusion protein. Three lines, C1, C2, and C3, showing the highest *CgS*_Δ90 _transcript- and CgS_Δ90 _protein levels were selected for further studies (Figure 2).

To increase the incorporation of free methionine into a storage protein, co-transformation experiment was carried out using two *Agrobacterium *strains. One of them contained a plasmid with the *CgS*_*Δ*90 _gene [12], while the other carried a construct for constitutive expression of the methionine-rich storage protein, 15-kD β-zein fused to 3HA [15]. This protein naturally accumulates in ER-derived protein bodies, and is stable in seed- and non-seed tissues [16-19]. After transformation, 25 independent kanamycin-resistant potato lines were obtained. Co-transformed lines were selected by PCR analysis using *CgS*_*Δ*90_*- *and *zein*-specific primers. Six lines were identified in which both transgenes were present. Two of these lines, designated C+Z1 and C+Z2, expressed both transgenes at high levels (Figure 2). The anti-HA recognised the CgS_Δ90 _and zein fusion proteins both in leaves and tubers. The level of expression, however, was higher in leaves than in tubers (Figure 2).

### Phenotype of the transgenic plants

**Figure 3:**
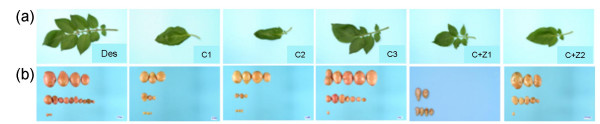
**Phenotypical characterisation of transgenic lines**. (a) Leaves and (b) tubers of the non-transformed control Désirée (Des), the *CgS*_*Δ*90 _transgenic lines, C1, C2, C3, and the *CgS*_*Δ*90_+*zein *double-transgenic lines, C+Z1 and C+Z2. Tubers represent the average yield/pot.

**Figure 4:**
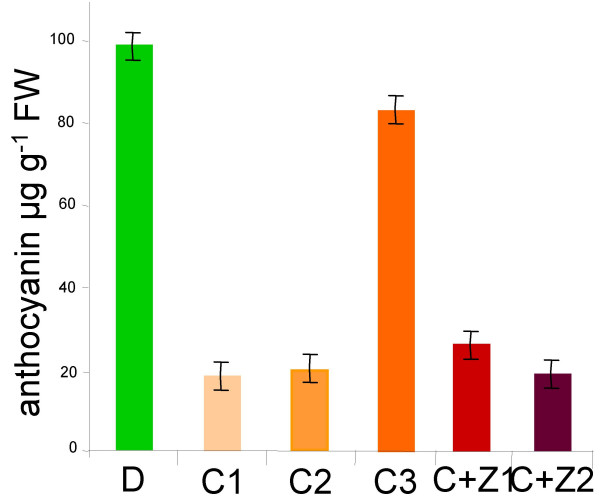
**Anthocyanin content of tuber skins**. Samples were derived from the non-transformed control Désirée (D), the *CgS*_*Δ*90 _transgenic lines, C1, C2, C3, and the *CgS*_*Δ*90_+*zein *double-transgenic lines, C+Z1 and C+Z2. Skins were collected from 3 × 3 tubers/line. Data are the mean ± SD. Cyanidin 3-*O*-glucoside chloride (Fluka) was used for calibration.

It was shown that modification of the methionine pathway by over-expressing the TS protein in potato caused phenotypic changes [4]. To investigate the effect of expressing the CgS_Δ90 _protein on the phenotype of potato non-transformed Désirée plants and the selected transgenic lines were transferred into soil and cultivated under greenhouse conditions. After six weeks, the plant material was evaluated and scored based on macroscopic phenotypic alterations. Transgenic line C3 was phenotypically almost indistinguishable from the non-transformed plants. The other 26 lines, however, exhibited serious alterations such as severe growth retardation and changes in leaf architecture. The strongest phenotypical alterations were displayed by lines C1 and C2 with no composite leaves at all. Lines C+Z1 and C+Z2 developed composite leaves, which, however, were smaller than those of the wild-type plants (Figure 3a). Due to severe growth retardation, a 40- to 60% reduction in tuber yield was also found (Figure 3b). Interestingly, we observed that the colour of the tubers' skin was also changed. The amounts of anthocyanin pigments were measured spectrophotemetrically in the skin of the non-transformed Désirée and its transgenic derivatives. Except of line C3, which was similar to the non-transformed control, a 4- to 5-fold reduction in the anthocyanin content of the skin of transgenic tubers was found compared to the control (Figure 4).

### Effect of co-expression of CgS_Δ90 _and the 15-kD β-zein on the free and protein-bound amino acid content of tubers

**Figure 5:**
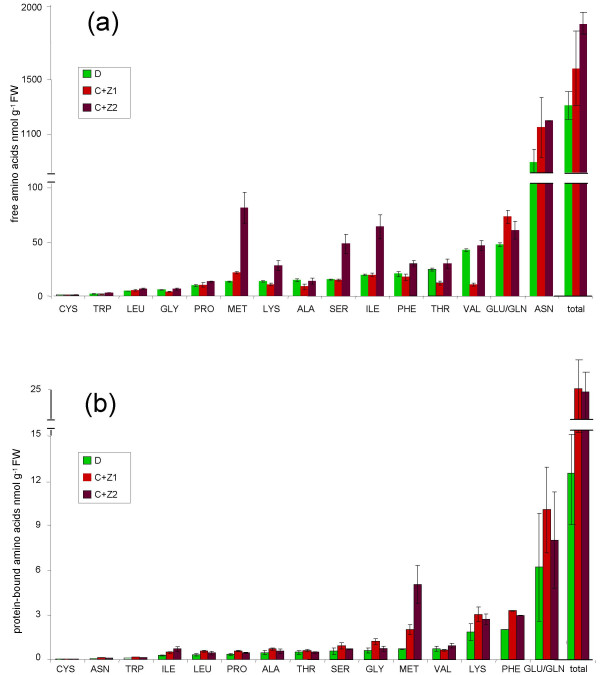
**Free and protein-bound amino acid content of tubers**. (a) Free amino acid content of tubers derived from the non-transformed control Désirée (D) and the *CgS*_*Δ*90_+*zein *double-transgenic lines, C+Z1 and C+Z2. Tubers were freshly harvested from four-month-old plants grown in soil in the greenhouse. Data were obtained from 2 × 3 peeled tubers/line measured in three parallels. Data are the mean ± SD. Differences in total free amino acid contents are significant at *P *= 0.01 (*t *probe) for the transgenic line C+Z2 compared to the non-transformed control. Differences in the amounts of amino acids other than methionine, serine, and isoleucine were not repeatable in consecutive experiments. (b) Amino acid composition of ethanol-soluble proteins of tubers derived from the non-transformed control Désirée (D) and the *CgS*_*Δ*90_+*zein *double-transgenic lines, C+Z1 and C+Z2. Data were obtained from the same set of tubers used for determination of free amino acids. Data are the mean ± SD. Differences in total protein-bound amino acid content of ethanol-soluble protein fractions are significant at *P *= 0.01 (*t *probe) for both transgenic lines compared to the non-transformed control. Glutamine and glutamic acid can not be separated by the method we used for amino acid analysis.

To investigate whether simultaneous expression of CgS_Δ90 _and zein changes the amino acid content of tubers, extracts of tuber flesh were analysed using a technology based on gas chromatography-mass spectrometry (GC-MS). The results are shown in Figure 5. We found that the amount of free methionine increased almost 2-fold in line C+Z1 and 6-fold in line C+Z2. In addition, the amount of serine and isoleucine was also 2- to 3-fold higher in C+Z2 tubers compared to wild-type. Elevated levels of these two amino acids, however, were not observed in the C+Z1 line. Although some variation in the levels of individual amino acids was observed in a fully independent second and third experiment, the changes detected in methionine, serine and isoleucine content were always repeatable. The total free amino acid content of line C+Z1 was not significantly different from that of the control, while a 1.4-fold increase was found in line C+Z2 (Figure 5a).

The protein content of tubers was also analysed. Because the 15-kD β-zein is an ethanol-soluble protein, the amino acid constitutions of proteins were determined not only in the water-, but also in the ethanol-soluble fraction. In the water-soluble fraction no significant difference between the transgenic lines and the control was detected (data not shown). In contrast, the amount of methionine in the ethanol-soluble fraction was much higher in the double transgenic than in the control plants, and, in particular, line C+Z2 displayed an about 2-fold higher level than line C+Z1. In sum, expression of zein resulted in an approximately 2-fold increase in the total ethanol-soluble protein content of tubers (Figure 5b).

### Effect of CgS_Δ90 _expression on phenylalanine ammonia lyase (*PAL*) mRNA levels

**Figure 6:**
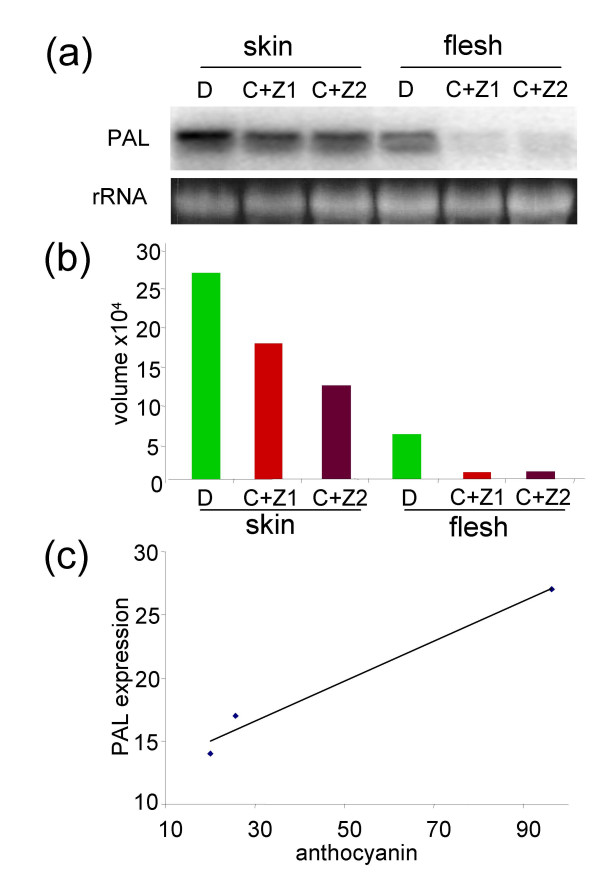
***PAL *expression in C+Z transgenic tubers**. (a) *PAL *mRNA levels detected by RNA gel blot analysis. Ethidium-bromide-stained rRNA bands are shown in the lower panel as loading controls. (b) Quantitative evaluation of hybridisation signals by phosphorimage analysis. Volume means the integrated intensity of all the pixels in the spot excluding the background. (c) Correlation between the anthocyanin content and level of *PAL *expression in tuber skin. Correlation coefficient *r *= 0.987931. The relationship is linear at *P *< 0.05.

We have found that anthocyanin content of the transgenic potato tubers expressing CgS_Δ90 _at high level was strongly reduced compared to the wild-type (Figure 4). Anthocyanins are flavonoid-type secondary metabolites whose synthesis starts with a reaction catalysed by PAL. It is known that the transcription of *PAL *has a differential regulation during plant development and by environmental cues [[20], and references therein]. We assumed that a reduction in *PAL *transcription could abolish the synthesis of anthocyanins and tested the *PAL *mRNA levels in both tuber skin and flesh on RNA-blots (Figure 6a). The *PAL *transcript level was four times higher in skin than in flesh in wild type tubers. The amount of *PAL *mRNA was 1.5- and 2-fold lower, respectively, in C+Z1 and C+Z2 tuber skins than in the control. A 7-fold decrease in the amount of *PAL *mRNA was detected in tuber flesh of lines C+Z1 and C+Z2 compared to the control (Figure 6b). There was a positive correlation between the anthocyanin content and level of *PAL *expression in tuber skin (Figure 6c).

## Discussion

### Expression of CgS_Δ90 _causes phenotypical changes in potato

In order to obtain potato plants with high methionine content in their tubers, we co-expressed the *CgS*_*Δ*90 _and the *15-kD β-zein *genes in the Désirée variety. We have found that the soluble methionine content of the obtained transgenic tubers was 2- to 6-fold higher compared to the wild-type, and that the transgenic plants displayed abnormal phenotypes, such as altered leaf morphology and growth, and changes in skin colour of tubers. Di et al. [7] expressed the full-length CgS of *Arabidopsis *in potato, which also resulted in a maximum of 6-fold elevation of methionine accumulation in tubers. All plants expressing the full-length CgS were phenotypically normal and indistinguishable from the non-transformed potato plants while our lines expressing CgS_Δ90 _alone or in combination with zein were phenotypically abnormal. It was demonstrated earlier that *Arabidopsis *and the T_1 _generation of transgenic tobacco plants over-expressing CgS_Δ90 _display an abnormal phenotype including stunted growth, slow development rate, narrow and curly leaves, and, in tobacco only, a high emission rate of the methionine catabolic product, dimethylsulfide [6,12]. Activity of the full-length CgS of *Arabidopsis *is regulated by methionine via SAM [9-11], while that of the potato is not [4,14]. Thus we concluded that irrespective of the mode of regulation of the endogenous CgS over-expression of CgS_Δ90 _results in phenotypic aberrations.

### Expression of CgS_Δ90 _alters the amino acid composition of tubers

**Figure 7:**
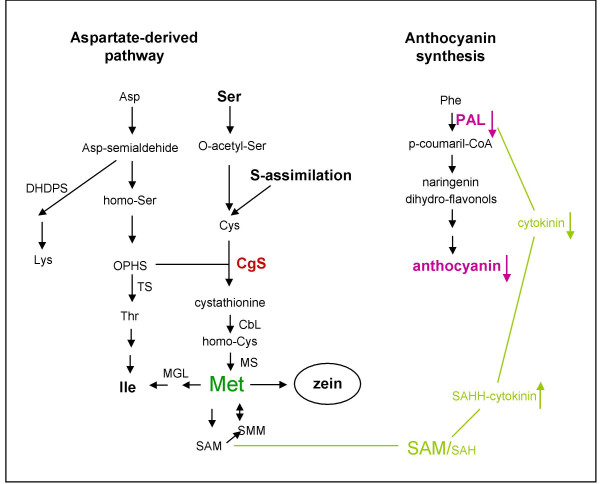
**Pathways of biosynthesis of the aspartate family of amino acids, including sulphur assimilation, and the pathway of anthocyanin synthesis**. The hypothetical connection between the methionin and anthocyanin synthesis is indicated in light green. Arrows in colour depict the effect of enhanced methionin synthesis. DHDPS, dihydrodipicolinate synthase; OPHS, *O*-phospho-homoserine; TS threonine synthase; CgS, cystathionine γ-synthase; CbL, cystathione β-lyase; MS, methionine synthase; MGL, methionine γ-lyase; SAM, *S*-adenosyl-L-methionine, SMM, *S*-methylmethionine; SAH, *S-*adenosylhomocysteine; SAHH, *S*-adenosylhomocysteine hydrolase; PAL, phenylalanine ammonia-lyase.

Methionine belongs to the aspartate family of amino acids (Figure 7). Interestingly, the C+Z2 tubers, which accumulated six times more soluble methionine than the control tubers, had 2- to 3-fold higher isoleucine and serine content too. Isoleucine is derived from threonine by deamination, and serine, which is converted to *O*-acetylserine, is important in sulphur assimilation (Figure 7). Thus it is likely that a fine mechanism of regulation exists, keeping the balance between the rate of synthesis of certain end- and start compounds in the aspartate-derived amino acid metabolism of potato. Rébeillé et al. [21] demonstrated that the expression of methionine γ-lyase (MGL) was induced in *Arabidopsis *cells in response to high methionine levels and that the production of *S*-methylcysteine and isoleucine was directly associated to the function of MGL (Figure 7). This step in methionine catabolism may also exist in potato and is responsible for the increased level of isoleucine in C+Z2 tubers.

In contrast, no significant difference in the levels of amino acids belonging to the aspartate family was observed between the wild-type and C+Z1 tubers. A huge variation depending on the intensity of transgene expression was detected also in the amino acid content of the leaves and siliques of transgenic *Arabidopsis *plants co-suppressed for CgS [5].

### Zein accumulates in the C+Z transgenic potato plants

The 15-kD β-zein protein of maize has already been successfully expressed in tobacco and alfalfa. Expression of zein did not result in phenotypical changes in transgenic plants [15-18]. Compared with plants expressing zein alone, transgenic alfalfa plants co-expressing zein and CgS contained enhanced level of zein concurrently with a reduction in the soluble methionine content [22]. To some extent, similar effect was detected in tobacco, in which, however, even the reduced amount of soluble methionine was accompanied by an abnormal phenotype [15]. We have observed similar phenomenon in potato. Co-expression of the 15-kD β-zein could not overcome the phenotypical changes caused by CgS_Δ90 _in potato.

Golan et al. [15] reported that expression of either CgS, or zein, or both resulted in increased amount of methionine incorporated in the water-soluble protein fraction of tobacco leaves. Major differences in the composition of water-soluble seed storage proteins were also reported for transgenic rice seeds expressing a methionine-rich storage protein [23]. In contrast, we found no alteration in the amino acid content of the water-soluble proteins in the C+Z potato tubers while a 2- to 6-fold increase in methionine content was observed in the zein-containing ethanol-soluble protein fraction. Considering, however, that 99% of tuber proteins are water-soluble (data not shown) the nutritional value of tubers could not be significantly improved by the 2-fold increase detected in the amount of ethanol-soluble proteins.

### Expression of CgS_Δ90 _reduces the anthocyanin content of tubers

Tubers of the potato cv. Désirée are characterised by red skin caused by the accumulation of anthocyanin pigments. Surprisingly, we have found that high constitutive expression of *CgS*_*Δ*90 _results in a 4- to 5-fold reduction in anthocyanin level of the tuber skin.

The anthocyanins are exclusively accumulated in the vacuole, although they are synthesised in the cytosol in the phenylpropanoid biosynthesis pathway that is initiated by deamination of phenylalanine catalysed by PAL (Figure 7). It is known that several environmental factors including light, wounding, or pathogen attack can affect anthocyanin levels of tubers [24]. Previous publications reported that repression of 14-3-3 protein synthesis or that of the chalcone synthase, chalcone isomerase, and dihydroflavonol reductase, enzymes involved in anthocyanin synthesis consecutive to PAL, decreased the amount of anthocyanins present in tuber skin [25,26]. We found a 7-fold reduction in the amount of *PAL *mRNA in flesh and a 1.5- to 2-fold reduction in skin of C+Z tubers compared to controls. In all species analysed to date, the common denominators in the regulation of anthocyanin biosynthesis genes are transcription factors with MYB or HLH domains and a WD40 protein in mutual interaction [27]. Co-ordinated, sucrose-specific modulation of the anthocyanin pathway is evidenced in *Arabidopsis*. However, this is likely achieved through the last few steps and does not influence *PAL *transcription [28]. *Arabidopsis *plants treated with exogenous cytokinins accumulate anthocyanin pigments. Deikman and Hammer [29] reported that PAL is controlled by the cytokinin benzyladenine posttranscriptionally. In contrast, using another cytokinin, isopentenyl adenosine, for treatment of *Arabidopsis *plants Németh et al. [30] detected higher amount of *PAL *mRNA in treated than in control plants. Assuming that transcriptional regulation of *PAL *by cytokinins exists in potato, a reduction in cytokinin level may explain the reduced amounts of *PAL *mRNA in the C+Z transgenic tubers compared to controls. The cytokinin-binding protein *S-*adenosylhomocysteine hydrolase (SAHH) that regulates biological methylation reactions through a control of the intracellular SAM/SAH ratio [2] may contribute to the alteration of cytokinin level in C+Z transgenic plants. Previously, it was found that anthocyanins accumulate in sulfur-starved *Arabidopsis *plants. The SAM decreased (3–30-fold) under sulphur deficiency, whereas SAH remained unchanged, the SAM/SAH ratio decreased accordingly [31,32]. Analysis of antisense SAHH tobacco plants showed that they contained excess levels of cytokinin [33]. High activity of the CgS_Δ90 _may increase the SAM level and SAHH activity reducing thereby the cytokinin level and transcription of *PAL *gene. Further biochemical and transcriptional investigations would help to elucidate the current hypothetical model (Figure 7), including the possible involvement of cytokinins in regulation of *PAL *expression in potato tubers.

## Conclusion

The free and protein-bound methionine content of tubers can be enhanced by co-expression of the CgS_Δ90 _and the methionine-rich storage protein, 15-kD β-zein. However, this elevation in methionine content is not high enough to significantly improve the nutritional value of tubers. The high level of CgS_Δ90 _expression can alter not only the methionine content of tubers but also the amount of other amino acids belonging to the aspartate family. This finding suggests that a fine regulatory mechanism exists between the pathways related to methionine synthesis and catabolism. Expression of CgS_Δ90 _has a pleiotropic effect on growth and yield of potato, and the anthocyinin content of tubers. The reduction in anthocyanin content may be explained by the reduction of mRNA levels of PAL, the first enzyme in the synthesis of anthocyanin pigments. Since there is no visible change in the phenotype of the arial parts of the potato plants repressed in other enzymes of anthocyanin biosynthesis than PAL [25,26] it is feasible that the growth retardation of CgS_Δ90_-expressing plants is not due to the reduced amounts of anthocyanins. Although, further investigations are needed to recover the signal transduction pathway between the amino acid and phenylpropanoid biosynthesis, to our knowledge this is the first report showing a linkage between methionine synthesis and *PAL *expression.

## Methods

### Plant material, growth conditions and transformation

*Solanum tuberosum *cv. Désirée was vegetatively propagated from single-node stem segments in tissue culture at 24°C under a 16 h light/8 h dark regime on MS or RM medium [34]. Transgenic potato lines were generated by leaf transformation according to Dietze et al. [35]. *Agrobacterium tumefaciens *strain C58C1 containing pGV2260 [36] was grown by standard techniques. The binary plasmid pZP111 carrying the deleted form (Δ358–447 bp) of *CgS *[12] and the same plasmid with a *15-kD β-zein *insert [15] were introduced into *Agrobacterium *by triparental mating [37]. Co-transformation was carried out by simultaneous infection of leaf explants by the two *A. tumefaciens *strains in equal cell density and volume. Six-week-old transgenic and control plants were transferred from tissue culture to pots and grown further under greenhouse conditions at 18–28°C for molecular and metabolite analysis as well as for yield tests. Tuberisation of 30 plants/line was tested in three independent experiments.

### Molecular biological techniques

Regenerated plants were screened for insertion of the transgene(s) by polymerase chain reaction. Genomic DNA was prepared from leaves as described by Shure et al. [38]. The 5'-gcaacatcggtgttgcacag-3'/5'-cagtgcttgcacacatccca-3' and the 5'-gatggtcatcgtctcgtcg-3'/gtagtagggcaggaatggca-3' primer pairs were used to test the presence of the *CgS*_*Δ*90 _and the *15-kD β-zein *genes in the potato genome, respectively.

Expression of the genes was tested by RNA-blot analysis. Total RNA was extracted from leaves using the method of Stiekema et al. [39] and separated on formaldehyde-agarose gel according to Logemann et al. [40]. Blotting and hybridisation was carried out under stringent conditions according to Sambrook et al. [41]. Radioactive DNA probes were generated by PCR, using the above described primers and ^32^P-labelled dCTP from the *CgS*_Δ90 _and *15-kD β-zein *cDNA as templates. The phenylalanine ammonia-lyase cDNA [NCBI: BE344057] was labelled by the random priming method [41].

### Analysis of anthocyanin pigments

Concentration of the anthocyanin pigments was measured with the simplified method of Toguri et al. [42]. Freshly harvested potato tubers of four-month-old plants grown in soil in greenhouse were peeled. Anthocyanin pigments were extracted from 1 g skin with 10 ml 1% HCl in methanol overnight at 4°C. Concentrations of the chloride forms of the anthocyanin pigments were determined spectrophotometrically by measuring the absorbance at 540 nm.

### Extraction of water- and ethanol-soluble protein fractions

100 mg leaf or tuber tissue was grinded in liquid nitrogen. The obtained powder was vigorously mixed with 400 μl of 0.1 M Na-phosphate buffer, pH 7.8, followed by centrifugation at 13000 rpm for 30 min at 4°C. Concentration of total water-soluble proteins in the supernatant was determined by the method of Bradford [43]. The ethanol-soluble protein fraction was extracted from the pellet with 400 μl solution containing 70% ethanol and 1% 2-mercapto-ethanol by incubation at 65°C for 30 min with shaking at 1000 rpm. Extracts were centrifuged at 13000 rpm for 30 min at 4°C.

### Protein-blot analysis

An aliquot containing 50 μg protein from the water- or ethanol-soluble fraction was dried. The dried material was dissolved in 10 μl loading buffer, and proteins were resolved by SDS-PAGE on 14% polyacrylamide gel for 250 Vh. After separation, proteins were transferred to nitrocellulose membrane and screened with Mono HA11 (Covance) using the HRP-based enhanced chemiluminescence (ECL) system (Amersham). HRP-linked anti-mouse IgG (Amersham) was used as secondary antibody.

### Amino acid analysis using GC-MS

For the analysis of free amino acids, polar metabolite fractions were extracted from 30 mg of potato tubers according to Nikiforova et al. [32].

For protein-bound amino acid analysis an aliquot containing 200 μg protein was transferred into a fresh Eppendorf tube from the water-soluble protein extract. The same volume was transferred from the ethanol-soluble fraction into a separate tube. Both samples were dried in a Speed-vac. 100 μl 6 N HCl solution was added to the dried samples, and incubated at 100°C with shaking at 500 rpm for 16 h. The solvent was then evaporated at 100°C. To the dry material 75 μl methanol, 25 μl chlorophorm and 100 μl water were added and mixed by vortexing. The mixtures were centrifuged at 13000 rpm for 5 min at 4°C, and the upper phase was dried in a Speed-vac.

Derivatization and metabolite analysis of soluble and protein-derived amino acids was carried out as described by Nikiforova et al. [32]. Ribitol was added as an internal standard. Samples were injected in splitless mode (1 μl/sample) and analysed in a quadrupole-type GC-MS system (Finnigan Trace/DSQ, Thermo Electron Corp.). The chromatograms and mass spectra were evaluated by using the XCALIBUR software (Thermo Electron Corp.) and the NIST 2.0 library.

## Authors' contributions

GD carried out protein extractions, protein-blot and GC-MS analysis; MK participated in potato transformation, selection and RNA-gel blot analysis of transgenic lines; ZB designed the experiments, co-ordinated the whole analysis and drafted the manuscript. All authors read and approved the final manuscript.
